# Learning dynamical information from static protein and sequencing data

**DOI:** 10.1038/s41467-019-13307-x

**Published:** 2019-11-26

**Authors:** Philip Pearce, Francis G. Woodhouse, Aden Forrow, Ashley Kelly, Halim Kusumaatmaja, Jörn Dunkel

**Affiliations:** 1 Department of Mathematics, Massachusetts Institute of Technology, Cambridge, Massachusetts 02139-4307 USA; 2 Mathematical Institute, University of Oxford, Andrew Wiles Building, Woodstock Road, Oxford, OX2 6GG UK; 3 Department of Physics, Durham University, South Road, Durham, DH1 3LE UK

**Keywords:** Computational biology and bioinformatics, Applied mathematics, Biological physics

## Abstract

Many complex processes, from protein folding to neuronal network dynamics, can be described as stochastic exploration of a high-dimensional energy landscape. Although efficient algorithms for cluster detection in high-dimensional spaces have been developed over the last two decades, considerably less is known about the reliable inference of state transition dynamics in such settings. Here we introduce a flexible and robust numerical framework to infer Markovian transition networks directly from time-independent data sampled from stationary equilibrium distributions. We demonstrate the practical potential of the inference scheme by reconstructing the network dynamics for several protein-folding transitions, gene-regulatory network motifs, and HIV evolution pathways. The predicted network topologies and relative transition time scales agree well with direct estimates from time-dependent molecular dynamics data, stochastic simulations, and phylogenetic trees, respectively. Owing to its generic structure, the framework introduced here will be applicable to high-throughput RNA and protein-sequencing datasets, and future cryo-electron microscopy (cryo-EM) data.

## Abstract (web summary)

Reconstructing system dynamics on complex high-dimensional energy landscapes from static experimental snapshots remains challenging. Here, the authors introduce a framework to infer the essential dynamics of physical and biological systems without need for time-dependent measurements.

## Introduction

Energy landscapes encapsulate the effective dynamics of a wide variety of physical, biological, and chemical systems^1,2^. Well-known examples include a myriad of biophysical processes^3–7^, multi-phase systems^2^, thermally activated hopping in optical traps^8,9^, chemical reactions^1,10^, brain neuronal expression^11^, cellular development^12–16^, and social networks^17^. Energetic concepts have also been connected to machine learning^18^ and to viral fitness landscapes, where pathways with the lowest energy barriers may explain typical mutational evolutionary trajectories of viruses between fitness peaks^19,20^. Recent advances in experimental techniques including cryo-electron microscopy (cryo-EM)^3,21,22^ and single-cell RNA-sequencing^23^, as well as new online social interaction datasets^24^, are producing an unprecedented wealth of high-dimensional instantaneous snapshots of biophysical and social systems. Although much progress has been made in dimensionality reduction^25–27^ and the reconstruction of effective energy landscapes in these settings^3,13,16,17,28^, the problem of inferring dynamical information such as protein-folding or mutation pathways and rates from instantaneous ensemble data remains a major challenge.

To address this practically important question, we introduce here an integrated computational framework for identifying metastable states on reconstructed high-dimensional energy landscapes and for predicting the relative mean first passage times (MFPTs) between those states, without requiring explicitly time-dependent data. Our inference scheme employs an analytic representation of the data based on a Gaussian mixture model (GMM)^29^ to enable efficient identification of minimum-energy transition pathways^30–32^. We show how the estimation of transition networks can be optimized by reducing the dimension of a high-dimensional landscape while preserving its topology. Our algorithm utilizes experimentally validated analytical results^8,9^ for transition rates^1,33–35^. Thus, it is applicable whenever the time evolution of the underlying system can be approximated by a Fokker–Planck-type Markovian dynamics, as is the case for a wide range of physical, chemical, and biological processes^1,34^.

Specifically, we illustrate the practical potential by inferring protein-folding transitions, state-switching in gene-regulatory networks, and HIV evolution pathways. Current standard methods for coarse-graining the conformational dynamics of biophysical structures^36,37^ typically estimate Markovian transition rates from time-dependent trajectory data in large-scale molecular dynamics (MD) simulations^38,39^. By contrast, we show here that protein-folding pathways and rates can be recovered without explicit knowledge of the time-dependent trajectories, provided the system is sufficiently ergodic and equilibrium distributions are sampled accurately. Furthermore, we show that the dynamics of state-switching or phenotype-switching in gene-regulatory networks^40^ can be inferred directly from static snapshots of protein abundances in regimes where deterministic modeling only captures a single steady state^41,42^. The agreement of our inferred results with two separate sets of time-dependent measurements suggests that the inference of complex transition networks via reconstructed energy landscapes can provide a viable and often more efficient alternative to traditional time-series estimates, particularly as new experimental techniques will offer unprecedented access to high-dimensional ensemble data.

## Results

### Minimum-energy-path network reconstruction

**Fig. 1:**
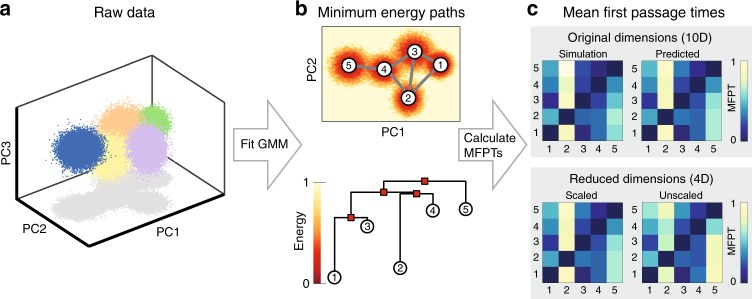
Inference scheme for estimating transition networks and mean first passage times (MFPTs). We apply the protocol to test data generated from a Gaussian Mixture Model (GMM; Supplementary Methods). **a** Inputs are the instantaneously measured data, sampled here from a ten-dimensional GMM with five Gaussians, plotted in the first three principal components (PCs); colors denote the Gaussian that a point was sampled from. **b** Top: a GMM is fit to the samples to construct the empirical probability distribution, which is then converted to the energy landscape using Eq. (1). Background color indicates the projection of the empirical energy landscape onto the first two PCs. Minimum-energy paths (MEPs, gray lines) between minima 1–5 on the landscape are calculated using the NEB algorithm (Supplementary Methods). Bottom: disconnectivity graph illustrating minima on the energy landscape (circles) and saddle points between them (squares). **c** A Markov state model (MSM) is constructed with transition rates given by Eq. (2) and solved to predict the MFPTs between discrete states (top right; Methods). MFPTs predicted by the MSM agree with direct estimates from Brownian dynamics simulations in the inferred energy landscape (top left; Supplementary Methods). MFPTs calculated in a reduced four-dimensional space using the scaling given in Eq. (3) recover the MFPTs accurately (bottom left). Without the appropriate scaling, the predicted MFPTs are inaccurate (bottom right).

The equilibrium distribution $p\left({\bf{x}}\right)$ of a particle diffusing over a potential energy landscape $E({\bf{x}})$ is the Boltzmann distribution $p({\bf{x}})=\exp \left[-E({\bf{x}})/{k}_{\text{B}}T\right]/Z,$ where ${k}_{\text{B}}$ is the Boltzmann constant, $T$ is the temperature, and $Z$ is a normalization constant. Given the probability density function (PDF) $p({\bf{x}})$, the effective energy can be inferred from

1 $$E({\bf{x}})=-{k}_{\text{B}}T\,{\mathrm{ln}}[p({\bf{x}})/{p}_{\max }],$$

where ${p}_{\max }$ is the maximum value of the PDF, included to fix the minimum energy at zero. Our goal is to estimate the MFPTs between minima on the landscape using only sampled data. We divide this task into three steps, as illustrated in Fig. 1 for test data (Supplementary Methods). In the first step, we approximate the empirical PDF by using the expectation maximization algorithm to fit a GMM in a space of sufficiently large dimension $d$ (Methods and Fig. 1a). Mixtures with a bounded number of components can be recovered in time polynomial in both the dimension $d$ and the required accuracy^43^. The resulting GMM yields an analytical expression for $E({\bf{x}})$ via Eq. (1).

In the second step, the inferred energy landscape $E({\bf{x}})$ is reduced to a minimum-energy-path (MEP) network whose nodes (states) are the minima of $E({\bf{x}})$ (Fig. 1b top). Each edge represents an MEP that connects two adjacent minima and passes through an intermediate saddle point (Fig. 1b). The MEPs are found using the nudged elastic band (NEB) algorithm^30,31^, which discretizes paths with a series of bead-spring segments (Supplementary Methods).

### Markov state model

Given the MEP network, the final step is to infer the rates for transitioning from a minimum $\alpha$ to an adjacent minimum $\beta$. Assuming overdamped Brownian dynamics, the directed transition $\alpha \to \beta$ can be characterized by the generalized Kramers transition rate^1^

2 $${k}_{\alpha \beta }=\frac{{\omega }_{\text{b}}}{2\pi \gamma }\frac{{\prod }_{i}{\omega }_{i}^{\alpha }}{\prod ^{\prime}_{i} {\omega }_{i}^{S}}\exp \left(-{E}_{\text{b}}/{k}_{\text{B}}T\right),$$

where $\gamma$ is the effective friction, ${E}_{\text{b}}$ is the energy difference between the saddle point $S$ on the MEP (over the energy barrier) and the minimum $\alpha$, ${\omega }_{i}^{\alpha }$ are the stable angular frequencies at the minimum $\alpha$, and ${\omega }_{i}^{S}$ and ${\omega }_{\text{b}}$ are the stable and unstable angular frequencies at the saddle, respectively. Equation (2) assumes isotropic friction but can be generalized to a tensorial form^1^ if anisotropies are relevant. In most practical applications, the error from assuming $\gamma$ to be isotropic is likely negligible compared with other experimental noise sources. In principle, Eq. (2) can be refined further by including quartic (or higher) corrections to the prefactor ${\omega }_{\text{b}}/\gamma$ to account for details of the saddle shape^1^. Such corrections can be significant for GMMs (Supplementary Methods).

Each edge $(\alpha \beta )$ has two weights, ${k}_{\alpha \beta }$ and ${k}_{\beta \alpha }$, assigned to it. The rate matrix $({k}_{\alpha \beta })$ completely specifies the Markov state model (MSM) on the network. Solving the MSM yields the matrix of pairwise MFPTs between states (Fig. 1c and Methods). In a simple two-state system, the MFPTs are determined up to a time scale by detailed balance, but for three or more states the influence of landscape topography and the associated state network topology (Methods) can lead to interesting hierarchical ordering of passage times. Identifying these hierarchies and ways to manipulate them is the key to controlling protein-folding or viral evolution pathways.

### Topology-preserving dimensionality reduction

To ensure that the inference protocol can be efficiently applied to larger systems with a high-dimensional energy landscape, we derive a general method for reducing the dimension $D$ of an energy landscape while preserving its topology. A PDF with $C$ well-separated Gaussians in $D$ dimensions can be projected onto the $d=C-1$ dimensional hyperplane spanning the Gaussian means using principal component analysis (PCA); projecting onto a hyperplane of dimension $d\, -\, 1$ risks losing information about the relative positions of the Gaussian means and, in general, does not allow a correct recovery of the MFPTs (Supplementary Methods). In practice, it suffices to choose $C$ to be larger than the number of energy minima if their number is not known in advance.

To preserve the topology under such a transformation—which is essential for the correct preservation of energy barriers and MEPs in the reduced-dimensional space—one needs to rescale GMM components in the low-dimensional space depending on the covariances of the Gaussians in the $D-d$ neglected dimensions (Fig. 1c). Explicitly, one finds that within the subspace spanned by the retained principal components (Supplementary Methods)

3 $$p({{\bf{x}}}_{D})=\sum _{i=1}^{C}{\phi }_{i}\ {p}_{i}^{d}({{\bf{x}}}_{d})\frac{\sqrt{{\mathrm{det}} \left(2\pi {\bf{U}}^{T}_{d}{{\mathbf{\Sigma }}}_{i}{{\bf{U}}}_{d}\right)}}{\sqrt{{\mathrm{det}} \left(2\pi {{\mathbf{\Sigma }}}_{i}\right)}}$$

as long as $p$ satisfies certain minimally restrictive conditions (Supplementary Methods). Here, ${{\bf{U}}}_{d}$ denotes the first $d=C-1$ columns of the matrix of sorted eigenvectors ${\bf{U}}$ of the covariance matrix of the Gaussian means, and ${\phi }_{i}$, ${p}_{i}^{d}$ and ${\mathbf{\Sigma} }_{i}$ are the mixing components, reduced-dimensional PDF, and the covariance matrix of each individual Gaussian in the mixture, respectively (Supplementary Methods). Neglecting the determinant scale factors in Eq. (3), as is often done when GMM models are fitted to PCA-projected data, leads to inaccurate MFPT estimates (Fig. 1c, bottom). It is noteworthy that Eq. (3) does not represent inversion of the transformation performed on the data by PCA, unless all $D$ dimensions are retained; if some dimensions are neglected, Eq. (3) represents a rescaling of the marginal distribution in the retained dimensions to reconstruct the PDF in the original dimension. In other words, the transition rates are best recovered from the conditional—not marginal—distributions, which are given by Eq. (3) up to a constant factor that does not affect energy differences.

Dimensionality reduction can substantially improve the efficiency of the NEB algorithm step as follows: when the MEPs in the reduced $d$-dimensional space have been computed, the identified minima and saddles can be transformed back into the original data dimension $D$ to calculate the Hessian matrices at these points, allowing Kramers’ rates to be calculated as usual (Fig. 1c and Supplementary Methods). Alternatively, in specific situations where the MEPs lie outside the hyperplane spanning the means (Supplementary Methods), the MEP in the reduced $d$-dimensional space can be transformed back to the $D$-dimensional space and can be used as an initial condition in that space, significantly reducing computational cost. These results present a step towards a general protocol for identifying reaction coordinates or collective variables for projection of a high-dimensional landscape onto a reduced space, while quantitatively preserving the topology of the landscape.

### Protein folding

To illustrate the vast practical potential of the above scheme, we demonstrate the successful recovery of several protein-folding pathways, using data from previous large-scale MD simulations^38^. The protein trajectories, consisting of the time-dependent coordinates of the alpha carbon backbone, were pre-processed, subsampled by a factor of 5, treated as a set of static equilibrium measurements, and reduced in dimension before fitting a GMM (Methods). As is typical for high-dimensional parameter estimation with few structural assumptions, the fitting error due to a finite sample size $n$ in $d$ dimensions scales approximately as $\sqrt{d/n}$ (Supplementary Methods); see refs. ^44–46^ for advanced techniques tackling sample-size limitations. Here, $d\,<\,10$ so the sample size $n \sim 1{0}^{5}$ suffices for effective recovery; indeed, our results were found to be robust for trajectories further subsampled by up to a factor of 25, leaving around 500 samples per Gaussian (Supplementary Fig. 3).

**Fig. 2:**
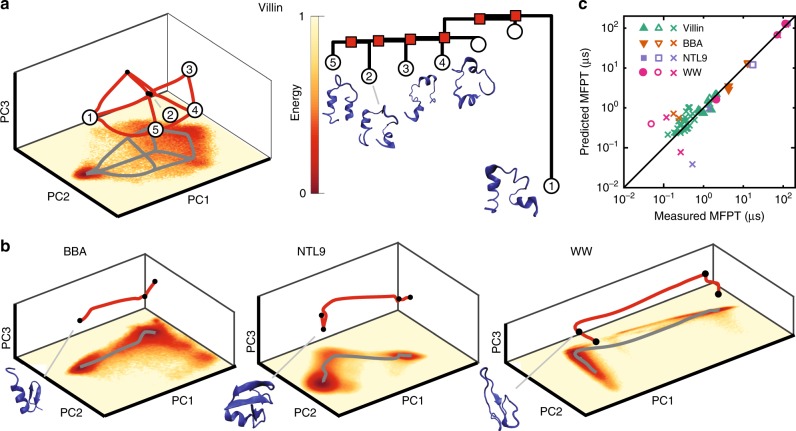
Reconstructed MEP networks for protein-folding transitions. We compare predicted MFPTs with direct estimates from molecular dynamics (MD) simulations (Supplementary Methods). **a** Left: low-energy states and transition network in the first three principal components (PCs) for Villin including predicted transition paths between states (red lines); bottom coloring shows two-dimensional projection of the empirical energy landscape onto the first two PCs. Right: associated disconnectivity graph and illustrations of the five lowest energy states, with state 1 corresponding to the folded state. **b** Low-energy states, transition paths, and empirical energy landscape for BBA, WW, and NTL9 proteins, and sketches of their folded states. **c** Predicted MFPTs agree well with estimates from MD simulations when energy minima are well separated and become less accurate for fast transitions with small MFPTs. Filled shapes correspond to transitions ending in the unfolded state and unfilled shapes correspond to transitions ending in the folded state (Supplementary Methods), for Villin, BBA, NTL9, and WW. Crosses correspond to transitions between intermediate states.

For each of the four analyzed proteins Villin, BBA, NTL9, and WW, the reconstructed energy landscapes reveal multiple states including a clear global minimum corresponding to the folded state (Fig. 2a, b). To estimate MFPTs, we determined the effective friction $\gamma$ in Eq. (2) for each protein from the condition that the line of best fit through the predicted vs. measured MFPTs has unit gradient. Although not usually known, $\gamma$ could in principle be calculated by incorporating time-dependent information from MD simulations or experimental data. Our MFPT predictions agree well with direct estimates (Supplementary Methods) from the time-dependent MD trajectories (Fig. 2c). Detailed analysis confirms that the MFPT estimates are robust under variations of the number of Gaussians used in the mixture (Supplementary Fig. 1). Also, the estimated MEPs are in good agreement with the typical transition paths observed in the MD trajectories (Supplementary Fig. 2).

### Gene-regulatory networks

**Fig. 3:**
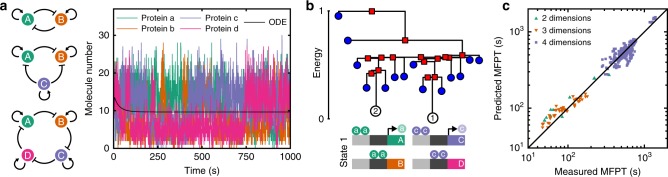
Reconstructed MEP networks for multistable gene-regulatory networks. We compare predicted MFPTs with direct estimates from stochastic simulations (Methods). **a** We performed simulations of three repressilator-type gene-regulatory network motifs with self-activation (left), consisting of two (top), three (middle), and four (bottom) genes, denoted A, B, C, and D. In the stochastic simulations (right), the numbers of each protein fluctuate between metastable states, but deterministic simulations of the system of ordinary differential equations (ODEs) at low molecule numbers are not able to identify the states, instead converging to a single steady state where all protein numbers are equal. **b** Disconnectivity graph and illustration of the identified lowest energy state for the four-dimensional system. In the lowest energy state (State 1), larger numbers of proteins a and c are present, leading to the activation of their associated genes and repressing the expression of proteins b and d. The next lowest energy state (State 2) is the converse scenario, with large numbers of proteins b and d present. **c** Predicted MFPTs agree well with those calculated from the time-dependent stochastic simulations for all three of the network motifs.

Next, we demonstrate the ability of our protocol to infer state-switching pathways in multistable gene-regulatory networks. Using a Gillespie stochastic simulation algorithm (SSA; Methods), we simulated three repressilator-type gene-regulatory network motifs^47^ with self-activation. Gene network motifs with features such as these have been studied extensively in recent years, owing to their ability to exhibit precise oscillations^48^ and to their possible importance in the determination of multiple cell fates^49^ in the appropriate parameter regimes, although the role of noise in such networks is not well understood. In our simulated gene networks, each gene encodes a protein that activates the expression of its associated gene and represses another, with $D=2$, 3, and 4 dimensions at low molecule numbers (Fig. 3a and Supplementary Methods). In each case, parameters were chosen to preclude oscillatory dynamics (Fig. 3a). The energy landscapes reconstructed from the simulation datasets in protein molecule-number space (with time-dependence removed) revealed multiple metastable states for each network (Fig. 3b and Supplementary Fig. 5). Broadly, we found each state to correspond to a mixture of low and high abundances for each separate protein, with the two most common states in $D=4$ dimensions consisting of two abundant and two depleted proteins (Fig. 3b). In agreement with previous studies^41,42^, the identified metastable states were not recovered from deterministic simulations of the governing ordinary differential equations (Supplementary Methods), but could only be identified directly from the stochastic data (Fig. 3a, b). We determined the effective friction $\gamma$ in Eq. (2) for each $D$ as in the protein example. The predicted MFPTs and MEPs between each metastable state were found to be accurate in comparison with time-dependent measurements (Fig. 3c and Supplementary Fig. 5b) and were robust to measurement noise typically encountered in single-cell sequencing (Supplementary Fig. 6). Our framework also correctly predicted MFPTs for a 5D asymmetric gene network (Supplementary Fig. 7). These results demonstrate the utility of our protocol for gene-regulatory network datasets and, more generally, energy landscapes in discrete spaces.

### Viral evolution

**Fig. 4:**
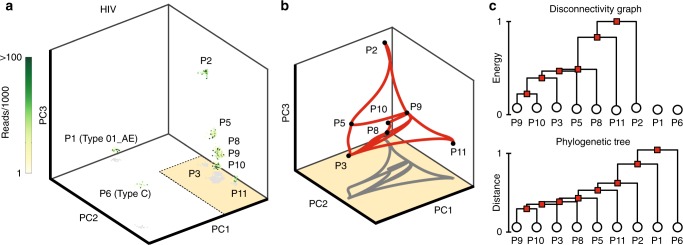
MEPs on viral fitness landscapes reconstructed from publicly available HIV sequencing data^50^. **a** Longitudinal samples of the HIV virus are binarized after multiple sequence alignment (Supplementary Methods) and plotted in the first three PCs. Samples of the same HIV subtype are closer in PC-space. Patient labels correspond to those used in ref. ^50^. **b** MEPs between minima corresponding to patients infected with Type B HIV, plotted in the first three PCs. Paths between minima indicate likely evolutionary pathways. Minima corresponding to patients with Type 01_AE and Type C HIV were unconnected to the other minima. **c** Disconnectivity graph for connected minima, where vertical evolution frequency is assumed to be proportional to the normalized energy barriers (top). The disconnectivity graph reproduces the majority of the structure of a distance-based phylogenetic tree (bottom), where the lengths of vertical lines are proportional to the Jukes-Cantor sequence distance (scaled to $[0,1]$).

As a final proof-of-concept application, we demonstrate that our inference scheme recovers the expected evolution pathways between HIV sequences as well as the key features of a distance-based phylogenetic tree (Fig. 4). To this end, we reconstructed an effective energy landscape from publicly available HIV sequences sampled longitudinally at several points in time from multiple patients^50^, assuming that the frequency of an observed genotype is proportional to its probability of fixation and that the high-dimensional discrete sequence space can be projected onto a continuous reduced-dimensional phenotype space (Fig. 4a and Supplementary Methods). First, a Gaussian was fit to each patient and then combined in a GMM with equal weights, to avoid bias in the fitness landscape towards sequences infecting any specific patient (Supplementary Methods). Thereafter, we applied our inference protocol to reconstruct the effective energy landscape, transition network (Fig. 4b), and disconnectivity graph (Fig. 4c), where each state is associated to a separate patient. As expected, states corresponding to patients infected with different HIV subtypes are not connected by MEPs (Fig. 4a, b). The disconnectivity graph reproduces the key features of a coarse-grained patient-level representation of the phylogenetic tree (Fig. 4c). Using our inference scheme, vertical evolution in the tree can be tracked along the MEPs in a reduced-dimensional sequence space (Fig. 4b). The energy barriers, represented by the lengths of the vertical lines in the disconnectivity graph (Fig. 4c), provide an estimate for the relative likelihood of evolution to fixation via point mutations between fitness peaks (energy minima). If mutation rates are known, the MEPs can also be used to estimate the time for evolution to fixation from one fitness peak to another^51^.

## Discussion

Finding the appropriate number of collective macro-variables to describe an energy landscape is a generic problem relevant to many fields. For example, although some proteins can be described through effective one-dimensional reaction coordinates^5,7,52,53^, the accurate description of their diffusive dynamics over the full microscopic energy landscape requires many degrees of freedom^54,55^. Whenever dynamics are inherently high-dimensional, topology-preserving dimensionality reduction can enable a much faster search of the energy landscape for minima and MEPs. In practice, data dimension is often reduced with PCA or similar methods before constructing an energy landscape^55–62^. The extent to which commonly used dimensionality reduction techniques alter MEP network topology or quantitatively preserve energy barriers is not well understood. Equation (3) suggests that reducing dimensions using PCA should not introduce significant errors if the variance of the landscape around each state (energy minimum) in the neglected dimensions is similar. For instance, we found that the protein-folding data could be reduced to five dimensions while maintaining accuracy (Supplementary Fig. 1), although additional higher energy states may become evident in higher dimensions. As an alternative to using Eq. (2) in the last stage of our approach, a method such as maximum caliber^63–65^, which does not take the derivatives of landscape topology into account, could be supplied with the sizes of the energy barriers and used to infer MFPTs. However, we found that owing to the dependence of the MFPTs on the prefactors in Eq. (2) for different transitions, this technique could not recover all transition rates accurately for either proteins or gene-regulatory networks (Supplementary Fig. 4). Overall, our theoretical results demonstrate the benefits of combining an analytical PDF with a linear dimensionality reduction technique so that the neglected dimensions can be accounted for explicitly.

Rapidly advancing imaging techniques, such as cryo-EM, will allow many snapshots of biophysical structures to be taken at the atomic level in the near future^3,21,22,28,66,67^. A biologically and biophysically important task will be to infer dynamical information from such instantaneous static ensemble measurements. The protein-folding example in Fig. 2 suggests that the framework introduced here can help overcome this major challenge; in principle, the framework requires only the pairwise distances between recognizable features of the protein as input (here we used the carbon alpha coordinates). Another promising area of future application is the analysis of single-cell RNA-sequencing data quantifying the expression within individual cells^23^. Related to this application, Fig. 3 demonstrates that our protocol recovers state-switching pathways in multistable gene-regulatory networks, which are thought to underlie cell-fate decisions. These results are most relevant in low-molecule-number regimes, in which noise is known to be an important factor^68^. In relevant recent work, an effective non-parametric energy landscape of single-cell expression snapshots was inferred using the Laplacian of a *k*-nearest neighbor graph on the data, allowing lineage information to be derived via a Markov chain^15^. The GMM-based framework here provides a complementary parametric approach for reconstructing faithful low-dimensional transition state dynamics from such high-dimensional data.

Furthermore, the proof-of-concept results in Fig. 4 suggest that our inference scheme for Markovian network dynamics can be useful for studying viral and bacterial evolution, which are often modeled as movements through a series of DNA or protein sequences^69^. The fitness landscape of an organism in sequence space is analogous to the negative of an effective energy landscape. The process of fixation by a succession of mutants in a population, whereby each mutant replaces the previous lineage as the population’s most recent common ancestor, has been modeled as a Markov process^70^. Successive sweeps to fixation have been observed in long-term evolution experiments, promising groundbreaking data for future analysis as whole-genome sequencing technologies improve^71^.

The inference protocol opens the possibility to analyze previously intractable multi-phase systems: many high-dimensional physical, chemical, and other stochastic processes can be described by a Fokker–Planck dynamics^1^, with phase equilibria corresponding to maxima of the stationary distribution. By taking near-simultaneous measurements of many subsystems within a large multistable Fokker–Planck system, the above scheme allows the inference of coexisting equilibria and transition rates between them. Other possible applications may include neuronal expression^11^ and social networks^17,24^, which have been described in terms of effective energy landscapes.

Although we focused here on normal white-noise diffusive behavior, as is typical of protein-folding dynamics, the above ideas can in principle be generalized to other classes of stochastic exploration processes. Such extensions will require replacing Eq. (2) through suitable generalized rate formulas, as have been derived for correlated noise^1^. Conversely, the present framework provides a means to test for diffusive dynamics: if the MFPTs of an observed system differ markedly from those inferred by the above protocol, then either important degrees of freedom have not been measured, the system is out of equilibrium on measurement time scales, or the system does not have Brownian transition statistics, necessitating further careful investigation of its time dependence.

By construction, the above framework is applicable to systems whose steady-state dynamics is approximately Markovian and can be described by a Fokker–Planck-type dynamics. This broad class includes thermal equilibrium systems as well as non-equilibrium systems that can be approximated by effective equilibrium theories^72,73^. However, such approximations can become inaccurate if probabilistic non-equilibrium fluxes dominate the system dynamics^74^. For example, reconstructing dynamical gene-expression information from static snapshots is sometimes possible in the presence of oscillatory dynamics caused by processes such as the cell cycle, but can fail for gene networks with large oscillations that are not orthogonal to the processes of interest^15^. Adapting the above protocol to reconstruct the dynamics in the latter case, and of far-from-equilibrium systems in general, will require incorporating more sophisticated theories that include time-resolved information^75–78^ and improved expressions for non-equilibrium transition rates^79^, and account for probabilistic fluxes^80^.

To conclude, the conformational dynamics of biophysical structures such as viruses and proteins, and the state-switching dynamics of noisy gene-regulatory networks, are characterized by their metastable states and associated transition networks, and can often be captured through Markovian models. Current experimental techniques, such as cryo-EM or RNA-sequencing, provide limited dynamical information. In these cases, transition networks must be inferred from static snapshots. Here we have introduced and tested a numerical framework for inferring Markovian state transition networks via reconstructed energy landscapes from high-dimensional static data. The successful application to protein-folding, gene-regulatory network, and viral evolution pathways illustrates that high-dimensional energy landscapes can be reduced in dimension without losing relevant topological information. In general, the inference scheme presented here is applicable whenever the dynamics of a high-dimensional physical, biological, or social system can be approximated by diffusion in an effective energy landscape.

## Methods

### Population landscapes

A GMM was used to represent the PDF, or population landscape, of samples. The PDF at position ${\bf{x}}$ of a GMM with $C$ mixture components in $d$ dimensions is

$$\begin{array}{rcl}p({\bf{x}}) 	=\sum _{i=1}^{C}{\phi }_{i}{p}_{i}({\bf{x}})\\ {p}_{i}({\bf{x}}) 	=\frac{\exp \left(-\frac{1}{2}{\left({\bf{x}}-{{\boldsymbol{\mu }}}_{i}\right)}^{T}{{\boldsymbol{\Sigma }}}_{i}^{-1}\left({\bf{x}}-{{\boldsymbol{\mu }}}_{i}\right)\right)}{\sqrt{\det \left(2\pi {{\boldsymbol{\Sigma }}}_{i}\right)}},\end{array}$$

where ${\phi }_{i}$ are the weights of each component, ${\boldsymbol{\mu} }_{i}$ are the means, and ${\boldsymbol{\Sigma} }_{i}$ are the covariance matrices. More details on GMMs and how they were fit to data are given in the Supplementary Methods.

### Mean first passage times

We form a discrete-state continuous-time Markov chain on states given by the minima of the energy landscape. For a pair of states $\alpha$ and $\beta$ directly connected by a minimum-energy pathway via a saddle, we approximate the transition rate $\alpha \to \beta$ by the Kramers rate ${k}_{\alpha \beta }$ in Eq. (2), whereas if $\alpha$ and $\beta$ are not directly connected we set ${k}_{\alpha \beta }=0$. Given these rates, the Markov chain has generator matrix ${M}_{\alpha \beta }$ where ${M}_{\alpha \beta }={k}_{\alpha \beta }$ for $\alpha \,\ne\, \beta$ and ${M}_{\alpha \alpha }=-{\sum }_{\beta :\beta \ne \alpha }{k}_{\alpha \beta }$. Then the matrix ${\tau }_{\alpha \beta }$ of MFPTs (hitting times) for transitions $\alpha \to \beta$ satisfies

$$\sum _{\gamma }{M}_{\alpha \gamma }{\tau }_{\gamma \beta }=-1\ \,\text{for}\,\ \alpha \ \ne\ \beta ,\quad {\tau }_{\alpha \alpha }=0.$$

### Protein data pre-processing

Protein-folding trajectories were obtained from all-atom MD simulations performed by D.E. Shaw Research^38^. Data were subsampled by a factor of 5 to reduce the size. For some proteins, residues at the flexible tails of proteins were removed from the dataset to reduce noise. Pairwise distances between carbon alpha atoms on the protein backbone were taken, with a cutoff of 6–8 Å, depending on the size of the protein; any distance above the threshold was taken to be equal to the threshold. This vector of pairwise distances was used as input to PCA, to reduce dimension. The first five principle components of the protein data were found to be sufficient for inference of energy landscapes and transition networks (Supplementary Fig. 1).

### Gene-regulatory network simulations

Gene-regulatory network motifs were simulated using a Gillespie SSA in the SimBiology toolbox in Matlab. A full list of the reactions simulated for each motif, as well as the values of the parameters used, is given in the Supplementary Methods and in the simulation code, which is available from Github (see Code availability).

### Reporting summary

Further information on research design is available in the Nature Research Reporting Summary linked to this article.

## Supplementary information

Supplementary Information

Reporting Summary

## Data Availability

Two publicly available datasets were used in this study. Protein-folding trajectories^38^ are available from D.E. Shaw Research (https://www.deshawresearch.com/). HIV sequences^50^ are available from https://hiv.biozentrum.unibas.ch/. Gene-regulatory network simulation data are available upon request, or can be generated by running the simulation code available from Github (see Code availability).

## Code availability

The source code used in this study to learn a dynamical transition network and mean first passage times from a Gaussian mixture model is publicly available from Github (https://github.com/philip-pearce/learning-dynamical). Also included are all data processing codes required to convert the raw data used in this study into the appropriate format and the simulation code for the gene-regulatory network simulations.
